# Analysis of Clinical Characteristics and Rehabilitation Outcomes in Elderly Patients with Parkinson’s Disease: A Retrospective Study

**DOI:** 10.3390/geriatrics10060163

**Published:** 2025-12-05

**Authors:** Toshiya Shimamoto, Yohei Misumi, Katsuhisa Uchino, Akira Mori, Takuya Motoshima, Makoto Uchino, Mitsuharu Ueda

**Affiliations:** 1 Department of Rehabilitation, Kumamoto Southern Regional Hospital, Kumamoto 861-4214, Japan; takuyamotoshima@outlook.jp; 2 Department of Neurology, Graduate School of Medical Sciences, Kumamoto University, Kumamoto 860-8556, Japan; misumiyohei@hotmail.co.jp (Y.M.); mitt@rb3.so-net.ne.jp (M.U.); 3 Department of Neurology, Kumamoto Southern Regional Hospital, Kumamoto 861-4214, Japan; katu-u@dp.u-netsurf.ne.jp (K.U.); akira5143@gmail.com (A.M.); uchinom1013@gmail.com (M.U.)

**Keywords:** Parkinson’s disease, aging, rehabilitation, sarcopenia, cognitive dysfunction

## Abstract

**Background**: Parkinson’s disease (PD) is a prevalent neurodegenerative disorder among the elderly, with its incidence increasing as the population ages. Despite the predominance of patients with PD aged 75 years and older in clinical settings, limited research has focused on their rehabilitation. This study aimed to compare the clinical characteristics and rehabilitation outcomes of elderly patients aged 75 years and older. **Methods:** A retrospective analysis was conducted on 141 patients with PD aged 65 years and older who underwent intensive inpatient rehabilitation. Patients were categorized into two subgroups: the young-old group (65–74 years, *n* = 58) and the old-old group (≥75 years, *n* = 83). The rehabilitation program included daily 2 h sessions, 6 days a week, combining physical, occupational, and speech–language–hearing therapies to enhance functional impairments and activities of daily living (ADL). Clinical characteristics and rehabilitation outcomes were compared between these groups. **Results:** The old-old group exhibited significantly higher rates of sarcopenia, higher Unified Parkinson’s Disease Rating Scale (UPDRS) scores, poorer balance scores and cognitive function, and lower ADL scores compared with the young-old group. However, both groups demonstrated significant improvements in UPDRS, Berg Balance Scale, 10 m walk test, and Functional Independence Measure scores, indicating enhanced motor function and ADL. **Conclusions**: Our retrospective study suggests that inpatient rehabilitation is associated with improvement in parkinsonism, motor symptoms, and ADL in patients with PD aged 75 years or older, highlighting the potential benefits of intensive rehabilitation even in advanced age. These findings underscore the need for prospective studies to confirm these effects. Trial registration: UMIN000056042 (last amendment 5 November 2024, retrospectively registered).

## 1. Introduction

Parkinson’s disease (PD) is a progressive neurodegenerative disorder characterized by motor disturbances, such as bradykinesia, resting tremor, rigidity, loss of postural reflexes, flexed posture, and difficulties with gait and balance, alongside non-motor disturbances, including cognitive and autonomic dysfunction [1,2,3]. PD is predominantly an age-related disease, affecting individuals over the age of 60 years. PD incidence is 14–19 per 100,000 person-years in developed countries and is higher among individuals over 65 years, with 160 per 100,000 person-years [4,5]. In Japan, the number of patients with PD aged ≥75 years in 2000 was 181,000, accounting for approximately 60% of the total population of patients with PD [6]. Previous studies have demonstrated that late-onset PD progresses more rapidly than early-onset PD, responds poorly to L-DOPA, and is often associated with prominent axial symptoms, such as postural and gait disturbances, and cognitive dysfunction [7,8,9,10,11]. These characteristics make disease management in older patients particularly challenging.

Pharmacotherapy is the mainstay symptomatic treatment for PD, but neurodegeneration involves non-dopaminergic brain regions, and dose-limiting side effects often restrict the effectiveness of these treatments [2]. Exercise has been associated with the modulation of neurotransmitters, changes in synaptogenesis, and increased cerebral blood flow, suggesting the potential for exercise-induced neuroplasticity [12]. Rehabilitation has been increasingly recognized as an effective complementary approach to pharmacotherapy, with various exercises, such as physiotherapy, gait training, and balance training, shown to provide notable benefits for motor and non-motor symptoms [13,14].

Although older patients aged ≥75 years with PD constitute the majority of those seen in daily clinical practice, there are limited reports focusing solely on this population. Given the global challenges posed by an aging population and the associated healthcare burdens, it is essential to understand the impact of aging on PD.

Therefore, the present study was aimed at clarifying the effects of intensive inpatient rehabilitation on motor, cognitive, and functional outcomes in older patients with PD and determining how age-related factors influence rehabilitation efficacy.

## 2. Materials and Methods

### 2.1. Participants

This retrospective study analyzed clinical data of 141 consecutive patients with PD who met the eligibility criteria for intensive inpatient rehabilitation at Kumamoto Southern Regional Hospital, between January 2019 and January 2021. All data were collected from our electronic medical record database.

All participants were admitted for PD-related motor and functional decline requiring intensive inpatient rehabilitation. Patients with comorbidities directly affecting motor function or rehabilitation outcomes—such as advanced orthopedic disease, significant cardiac disease, uncontrolled arrhythmias, severe pulmonary disease, severe dementia, or a history of stroke—were excluded. Additional exclusion criteria were atypical parkinsonian disorders (dementia with Lewy bodies, multiple system atrophy, progressive supranuclear palsy, and corticobasal degeneration), inability to walk ≥20 m even with walking aids, and transfer to another hospital due to acute clinical deterioration or death. The Joint Committee of Japan Gerontological Society and the Japan Geriatrics Society have defined age categories considering the aging population in developed countries, classifying individuals as young-old (65–74 years) and old-old (≥75 years) [15]. Patients were categorized into two age-based groups for comparative analysis of clinical characteristics and rehabilitation outcomes: the old-old group (*n* = 83; ≥75 years) and the young-old group (*n* = 58; 65–74 years).

### 2.2. Data Collection

Demographic data, including sex, age, age of onset, duration of disease, Hoehn and Yahr (H & Y) stage, body mass index (BMI), skeletal muscle index (SMI), blood test results, and medication use, were collected from institutional databases. Pre- and post-rehabilitation assessments were conducted to evaluate parkinsonism, motor and cognitive functions, and activities of daily living (ADL). Baseline assessments were performed within 48 h of admission, and follow-up assessments were conducted after completion of the standardized 4-week rehabilitation program. Information on comorbidities was also collected.

SMI was derived from body composition measured using segmental bioelectrical impedance (InBody S10, InBody Japan Inc., Tokyo, Japan). L-DOPA equivalent doses (LEDs) were calculated to standardize comparisons of daily anti-parkinsonian drug dosages [16]. Parkinsonism was assessed using the Unified Parkinson’s Disease Rating Scale (UPDRS), which comprises four sections: Part 1 (mental, behavioral, and mood), Part 2 (ADL), Part 3 (motor symptoms), and Part 4 (treatment complications). Subscores for tremors, rigidity, bradykinesia, and axial symptoms were extracted from Part 3 [17]. In Part 3, motor symptom subscores were extracted separately, and data were calculated: tremor, items 20 and 21; rigidity, item 22; bradykinesia, items 19, 23–26, and 31; and axial symptoms, items 18 and 27–30. The UPDRS was evaluated during the ON phase.

Motor function was assessed using the Berg Balance Scale (BBS) and 10-Meter Walking Test (10MWT). The BBS measures static and dynamic balance ability through 14 tasks, each rated on a scale of 0–4 points, with lower total scores indicating greater disease severity [18]. The 10MWT is used to evaluate gait velocity and identify changes in gait velocity in response to interventions in individuals with PD [19].

Cognitive function was assessed using the Montreal Cognitive Assessment Japanese (MoCA-J), a validated screening tool for mild cognitive impairment. The MoCA provides an assessment of global cognitive function, with scores ranging from 30 (normal) to 0 (most severe) [20]. Functional independence was evaluated using the Functional Independence Measure (FIM), an 18-item ordinal scale that assesses the level of disability and changes in status after interventions. Scores range from 18 (complete dependence) to 126 (complete independence), with each item rated on a scale of 1 to 7 [21].

To support interpretation of changes, previously reported Minimal Clinically Important Difference (MCID)/Minimal Detectable Change (MDC) values were referenced: UPDRS Part 3 (2.5–10 points), UPDRS Part 2 (2–3 points), UPDRS total (4–9 points), gait speed (0.05–0.22 m/s), and BBS (MDC95 = 5 points). PD-specific MCID/MDC values for MoCA and FIM are not established [22,23,24,25].

### 2.3. Rehabilitative Interventions

All patients participated in a standardized, multidisciplinary intensive rehabilitation program consisting of 2 h per day, 6 days per week, for 4 weeks. Physical therapy focused on gait training, balance and postural exercises, lower-limb strengthening, and mobility practice. Occupational therapy targeted ADL, upper-limb coordination, and fine motor control. Speech therapy included speech articulation, voice training, and cognitive-linguistic exercises.

Disease-specific programs such as LSVT-BIG and LSVT-LOUD were not routinely implemented during the study period.

### 2.4. Statistical Analysis

Continuous variables were summarized using means and standard deviations, while categorical variables were reported as proportions. The Kolmogorov–Smirnov test was used to assess the normality of data distribution. Group comparisons were performed using Student’s *t*-test, the Mann–Whitney U test, and the chi-square test for categorical variables. As applicable, rehabilitation outcomes for parkinsonism, motor and cognitive functions, and ADL were analyzed using paired *t*-tests and Wilcoxon signed-rank tests. Differences in rehabilitation outcomes between the two groups were evaluated using analysis of covariance (ANCOVA) with baseline values of each outcome, sex, H & Y stage, and LEDs as covariates.

Statistical analyses were conducted using JMP version 14 (SAS Institute, Cary, NC, USA), with statistical significance at *p* < 0.05.

## 3. Results

### 3.1. Clinical Characteristics

The mean age ± standard error was 79.0 ± 3.6 years in the old-old group and 70.1 ± 3.1 in the young-old group. Table 1 summarizes the clinical characteristics of both groups. There were no statistically significant differences in sex, disease duration, H & Y stage, BMI, number of medications, or daily LEDs between the groups. However, the old-old group exhibited a significantly lower SMI (*p* = 0.029) and grip strength (*p* = 0.043) and a higher prevalence of sarcopenia (59.0%) than the young-old group.

At baseline, only mild to moderate conditions were present due to the exclusion of severe disabling comorbidities at baseline. The most common comorbidities were controlled hypertension (36.8% in the young-old group vs. 45.2% in the old-old group), diabetes mellitus (26.3% vs. 16.4%), mild cardiovascular disease (20.5% vs. 22.8%), and dyslipidemia (32.9% vs. 36.8%).

Regarding parkinsonism, the UPDRS Part 2 (*p* = 0.007) and axial symptom subscores (*p* = 0.012) were significantly worse in the old-old group. Measures of motor and functional performance, including the BBS, 10MWT, MoCA-J, and FIM scores, were significantly lower in the old-old group than in the young-old group (*p* = 0.002, *p* < 0.001, *p* < 0.001, and *p* < 0.001, respectively). Serum albumin levels and estimated glomerular filtration rate (eGFR) were also significantly lower in the old-old group.

### 3.2. Outcomes of the Rehabilitation Intervention

Both groups demonstrated significant improvement in parkinsonism following the rehabilitation intervention. Improvements were observed in UPDRS Parts 1–3 and in total UPDRS scores (all *p* < 0.001, Table 2). Subscores for bradykinesia and axial symptoms also improved significantly in both groups. Motor and function performance measures―including BBS, 10MWT, and FIM―showed significant gains following rehabilitation. Daily LED exposure remained unchanged in both groups.

When interpreted against published MCID/MDC thresholds, improvements in UPDRS Part 3 (−3.1 points in the old-old group and −4.4 points in the young-old group) fell within the minimal-to-moderate clinically important range (approximately 2.5–5 points). Improvements in UPDRS Part 2 (−2.6 and −2.8 points) reached the commonly cited MCID of 2–3 points, indicating meaningful short-term gains in ADL. Improvements in UPDRS total scores (−9.6 and −11.5 points) also approached or exceeded the lower bound of reported MCID estimates (approximately 4–9 points). The increase in gait speed of about 0.1 m/s corresponded to clinically important differences reported in individuals with PD. In contrast, improvements in BBS (3.3–3.9 points) did not exceed the commonly cited MDC95 threshold of 5 points.

Between-group comparisons adjusted for baseline values, sex, H & Y stage, and LED (ANCOVA) revealed that the young-old group achieved greater improvements in UPDRS Part 3, bradykinesia. Axial symptoms, UPDRS total, BBS, and MoCA-J scores (all *p* < 0.05). No significant differences were found for UPDRS Parts 1, 2, and 4, 10MWT, FIM, or LED between the groups. These findings are summarized in Table 2.

## 4. Discussion

In the present study, individuals aged 75 years and above exhibited worse sarcopenia, nutritional status, renal function, motor symptoms, cognitive function, and ADL compared with those aged 65–74 years. These differences were observed despite the absence of significant differences in H & Y stage, disease duration, or daily LEDs between the groups. Intensive inpatient rehabilitation significantly improved motor symptoms and ADL in older patients with PD, including those aged ≥75 years. These findings support the growing evidence that structured rehabilitation can enhance functional outcomes in PD, even in advanced age.

Previous research, including a meta-analysis, has demonstrated that exercise interventions are effective for improving motor symptoms, balance and gait ability, and ADL in individuals with PD [13,14,26]. Additionally, physical activity, including physical and occupational therapy, has been reported to alleviate non-motor symptoms, including depression, apathy, fatigue, daytime sleepiness, sleep, and cognition [27]. Although few studies have specifically examined 75 years and above, several reports have shown that balance and resistance training improve parkinsonism and gait-related symptoms in older adults [28,29,30]. Our findings extend this evidence by demonstrating that individualized, multidisciplinary rehabilitation can yield meaningful improvements even in this late-older population. Intensive inpatient rehabilitation provides several advantages, including structured daily routines, multidisciplinary supervision, and psychosocial support, which may contribute to maintaining or improving ADL performance in older individuals with PD.

While rehabilitation significantly improved overall outcomes in young-old and old-old patients with PD, analyses adjusting for baseline values, sex, H & Y stage, and LED suggested that the effects of rehabilitation on UPDRS Part 3, bradykinesia, axial symptoms, BBS, and MoCA-J scores were slightly limited in the old-old group compared with those in the young-old group.

Several factors may explain the reduced responsiveness in the old-old group. First, sarcopenia and reduced muscle mass likely contributed to limited improvement. Muscle mass declines with age, particularly after 70 years, at a rate of 15% per decade [31], and sarcopenia is highly prevalent in PD [32], being associated with poorer exercise tolerance and reduced ADL independence [33,34,35]. Second, cognitive decline may affect the ability to learn and retain motor skills. The prevalence of cognitive dysfunction in PD has been estimated at 24–31% [36], with impairments reported in executive function, attention, memory, and visuospatial abilities [37]. A recent meta-analysis reported that exercise improves global cognitive function, including MMSE and MoCA scores, and executive function in individuals with PD [38]. In our study, MoCA-J scores were significantly improved in the young-old group but not in the old-old group, suggesting that potential for cognitive recovery may diminish with advancing age. These findings may reflect age-related physiological and neural limitations, such as impaired neural plasticity and decreased responsiveness of non-dopaminergic systems involved in gait and balance control [39,40,41,42,43]. Further studies are warranted to determine whether specific exercise modalities—such as resistance, dual-task, or cognitive training—can mitigate these limitations and optimize rehabilitation outcomes in advanced-age PD patients.

Changes in UPDRS Part 3, UPDRS Part 2, UPDRS total scores, and gait speed all met or approached established MCID thresholds, indicating that intensive inpatient rehabilitation can produce relevant gains in motor function, mobility, and ADL even among individuals aged 75 years and above. In contrast, improvements in BBS were below the MDC threshold, suggesting modest changes in balance. For MoCA-J and FIM, PD-specific MCID values remain uncertain, and the clinical interpretation of these measures should therefore be approached with caution.

This study had some limitations. First, it was a retrospective single-center study, non-randomized study, and therefore residual confounding cannot be fully excluded. Second, the study participants were limited to inpatients, and therefore the findings may not directly apply to outpatient or community-based rehabilitation. Third, the long-term effects and prognosis of rehabilitation were not evaluated.

## 5. Conclusions

This retrospective study suggests that intensive inpatient rehabilitation may improve motor, cognitive, and functional abilities in older patients with PD, including those aged ≥75 years. Further refinement of rehabilitation programs and prospective studies will be important to enhance rehabilitation effectiveness for an increasingly PD population.

## Figures and Tables

**Table 1:** Clinical characteristics of individuals in the old-old and young-old groups.

	Old-Old Group (≥75) (*n* = 83)	Young-Old Group (65–74) (*n* = 58)	*p*-Value
Age (years)	79.0 ± 3.7	70.1 ± 3.1	<0.001 *
Sex (female/male)	39/44	32/26	0.198
Disease duration (years)	7.8 ± 4.4	6.9 ± 4.6	0.145
H & Y stage	3.6 ± 0.6	3.4 ± 0.5	0.052
BMI (kg/m^2^)	21.6 ± 3.4	22.1 ± 3.8	0.819
SMI (kg/m^2^)	6.3 ± 1.0	6.6 ± 1.1	0.029 *
Grip strength (kg)	19.8 ± 5.3	23.4 ± 9.0	0.043 *
Sarcopenia (yes/no, prevalence %)	49/34, 59.0	18/40, 31.0	0.001 *
Blood test results	-	-	-
Alb (g/dL)	3.9 ± 0.4	4.1 ± 0.3	0.003 *
BUN/Cre	26.7 ± 7.3	26.9 ± 7.4	0.612
eGFR (mL/min/1.73 m^2^)	63.8 ± 15.1	69.3 ± 16.7	0.040 *
AST (U/L)	23.9 ± 8.2	25.7 ± 11.5	0.898
ALT (U/L)	19.5 ± 13.6	22.4 ± 12.8	0.122
ALP (U/L)	89.3 ± 31.5	88.3 ± 31.4	0.980
Number of drugs (excluding anti-parkinsonian drugs)	6.8 ± 2.3	5.9 ± 2.6	0.070
Daily LEDs (mg)	488.6 ± 244.2	504.9 ± 313.8	0.724
Parkinsonism	-	-	-
UPDRS Part 1	2.6 ± 2.1	2.2 ± 2.1	0.132
UPDRS Part 2	12.5 ± 6.0	9.7 ± 6.8	0.007 *
UPSRS Part 3	21.5 ± 9.6	20.1 ± 8.9	0.397
Tremor	0.9 ± 1.7	0.6 ± 1.0	0.879
Rigidity	3.3 ± 3.2	4.2 ± 2.7	0.061
Bradykinesia	10.5 ± 5.3	9.4 ± 5.2	0.223
Axial symptoms	6.7 ± 3.5	5.5± 3.5	0.012 *
UPDRS Part 4	2.5 ± 2.6	3.3 ± 2.9	0.252
UPDRS total	39.3 ± 17.2	35.3 ± 16.7	0.124
Motor function	-	-	-
BBS	42.6 ± 11.0	46.9 ± 9.8	0.002 *
10MWT (m/s)	0.9 ± 3.0	1.2 ± 0.4	<0.001 *
Cognitive function	-	-	-
MoCA-J	19.4 ± 5.0	22.6 ± 4.6	<0.001 *
ADL	-	-	-
FIM	99.7 ± 14.4	108.8 ± 16.0	<0.001 *

* Significant difference at *p* < 0.05. Data are expressed as mean ± standard deviation (SD) unless otherwise indicated. Abbreviations: H & Y, Hohen and Yahr; BMI, body mass index; SMI, skeletal muscle mass; Alb, albumin; BUN, blood urea nitrogen; Cre, creatinine; eGFR, estimated glomerular filtration rate; AST, aspartate aminotransferase; ALT, alanine aminotransferase; ALP, alkaline phosphatase; LED, L-DOPA equivalent dose; UPDRS, Unified Parkinson’s Disease Rating Scale; BBS, Berg Balance Scale; 10MWT, 10 m walking test; FIM, Functional Independence Measure; MoCA-J, Montreal Cognitive Assessment-Japanese version.

**Table 2:** Comparison of rehabilitation outcomes in individuals in the old and young-old groups.

	Pre-Intervention	Post-Intervention	Within Group Comparison from Pre to Post Intervention	Between Group Comparison of Changes from Pre to Post Intervention
			*p*-Value	
UPDRS Part 1				
Old-old group (≥75)	2.7 ± 2.1	1.7 ± 1.8	<0.001 *	0.802
Young-old group (65–74)	2.2 ± 2.1	1.4 ± 1.7	<0.001 *	
UPDRS Part 2				
Old-old group	12.1 ± 5.2	9.5 ± 5.3	<0.001 *	0.097
Young-old group	9.7 ± 6.8	6.9 ± 5.8	<0.001 *	
UPDRS Part 3				
Old-old group	21.6 ± 9.3	18.5 ± 8.0	<0.001 *	0.010 *
Young-old group	20.1 ± 8.9	15.7 ± 8.3	<0.001 *	
Tremor				
Old-old group	0.9 ± 1.7	0.6 ± 1.4	0.062	0.986
Young-old group	0.6 ± 1.0	0.5 ± 0.9	0.226	
Rigidity				
Old-old group	3.5 ± 3.1	3.4 ± 3.1	0.692	0.651
Young-old group	4.2 ± 2.7	4.0 ± 2.7	0.213	
Bradykinesia				
Old-old group	10.5 ± 5.2	9.3 ± 4.7	<0.001 *	0.042 *
Young-old group	9.4 ± 5.2	7.5 ± 4.9	<0.001 *	
Axial symptoms				
Old-old group	6.7 ± 3.5	5.5 ± 3.0	<0.001 *	0.023 *
Young-old group	5.5 ± 3.5	4.1 ± 3.3	<0.001 *	
UPDRS Part 4				
Old-old group	2.5 ± 2.6	2.2 ± 1.8	0.065	0.150
Young-old group	3.3 ± 2.9	2.9 ± 2.8	0.066	
UPDRS total				
Old-old group	39.1 ± 14.6	29.5 ± 12.8	<0.001 *	0.009 *
Young-old group	35.3 ± 16.7	23.8 ± 13.9	<0.001 *	
BBS				
Old-old group	42.7 ± 10.3	46.0 ± 8.9	<0.001 *	0.019 *
Young-old group	46.9 ± 9.8	50.7 ± 8.1	<0.001 *	
10MWT				
Old-old group	0.9 ± 0.3	1.0 ± 0.3	0.003 *	0.081
Young-old group	1.2 ± 0.4	1.3 ± 0.4	<0.001 *	
MoCA-J				
Old-old group	19.5± 5.4	20.5 ± 5.2	0.091	0.006 *
Young-old group	22.6 ± 4.6	24.1 ± 4.2	<0.001 *	
FIM				
Old-old group	99.5± 14.8	107.0 ± 13.6	<0.001 *	0.380
Young-old group	108.8 ± 16.0	114.6 ± 12.5	<0.001 *	
Daily LEDs (mg)				
Old-old group	488.6 ± 244.2	496.7 ± 237.4	0.439	0.435
Young-old group	504.9 ± 313.8	527.3 ± 296.0	0.171	

* Significant difference at *p* < 0.05. Data are presented as mean ± standard deviation (SD) unless otherwise specified. Abbreviations: UPDRS, Unified Parkinson’s Disease Rating Scale; BBS, Berg Balance Scale; 10MWT, 10 m walking test; MoCA-J, Montreal Cognitive Assessment-Japanese version; FIM, Functional Independence Measure; LED, L-DOPA equivalent dose.

## Data Availability

Data can be accessed upon reasonable request to the corresponding author due to ethical and privacy restrictions.

## Abbreviations

The following abbreviations are used in this manuscript:

PD	Parkinson’s disease
ADL	Activities of daily living
H & Y	Hohen and Yahr
BMI	Body mass index
SMI	Skeletal muscle index
LED	L-DOPA equivalent dose
UPDRS	Unified Parkinson’s Disease Rating Scale
BBS	Berg Balance Scale
10MWT	10-Meter Walking Test
MoCA-J	Montreal Cognitive Assessment Japanese
FIM	Functional Independence Measure
